# Comparison of COVID-19 and Influenza-Related Outcomes in the United States during Fall–Winter 2022–2023: A Cross-Sectional Retrospective Study

**DOI:** 10.3390/diseases12010016

**Published:** 2024-01-03

**Authors:** Hagit Kopel, Alina Bogdanov, Jessamine P. Winer-Jones, Christopher Adams, Isabelle H. Winer, Machaon Bonafede, Van Hung Nguyen, James A. Mansi

**Affiliations:** 1 Moderna, Inc., Cambridge, MA 02139, USA; hagit.kopel@modernatx.com; 2 Veradigm, Chicago, IL 60654, USA; alina.bogdanov@veradigm.com (A.B.); jessamine.winer-jones@veradigm.com (J.P.W.-J.);; 3 VHN Consulting Inc., Montreal, QC H2V 3L8, Canada; vhnguyen@vhnconsulting.com

**Keywords:** COVID-19, SARS-CoV-2, influenza, United States, hospitalization

## Abstract

Influenza and COVID-19 contribute significantly to the infectious disease burden during the respiratory season, but their relative burden remains unknown. This study characterizes the frequency and severity of medically attended COVID-19 and influenza during the peak of the 2022–2023 influenza season in the pediatric, adult, and older adult populations and characterizes the prevalence of underlying conditions among patients hospitalized with COVID-19. This cross-sectional analysis included individuals in the Veradigm EHR Database linked to Komodo claims data with a medical encounter between 1 October 2022 and 31 March 2023 (study period). Patients with medical encounters were identified with a diagnosis of COVID-19 or influenza during the study period and stratified based on the highest level of care received with that diagnosis. Among 23,526,196 individuals, there were more COVID-19-related medical encounters than influenza-related encounters, overall and by outcome. Hospitalizations with COVID-19 were more common than hospitalizations with influenza overall (incidence ratio = 4.6) and in all age groups. Nearly all adults hospitalized with COVID-19 had at least one underlying medical condition, but 37.1% of 0–5-year-olds and 25.0% of 6–17-year-olds had no underlying medical conditions. COVID-19 was associated greater burden than influenza during the peak of the 2022–2023 influenza season.

## 1. Introduction

Severe acute respiratory syndrome coronavirus 2 (SARS-CoV-2), the virus responsible for the coronavirus 2019 (COVID-19) respiratory infection, was first detected in Wuhan, China, in December of 2019 [1]. It quickly spread globally and has proven to be a persistent public health threat due in part to variants with enhanced transmissibility and pathogenicity [2]. In addition, previously widespread variants are rapidly displaced by new variants and subvariants that more effectively evade existing natural and vaccine immunity [3]. As a result, despite widespread immune exposure to the SARS-CoV-2 virus, test positivity for COVID-19 remains high and exceeded test positivity for influenza and respiratory syncytial virus during the majority of the last 12 months in the United States (US) [4]. Infection may result from a short but intense exposure or following prolonged or repeated exposure to a smaller dose over time, such as when SARS-CoV-2 is introduced within a household. One study found that while 75% of children had an asymptomatic infection, the secondary attack rate in the home was 57.7% [5].

Although COVID-19 is no longer considered a public health emergency [6], it continues to be the leading respiratory infectious disease causing hospitalizations in the US [4,7,8]. Previous research has shown that COVID-19 disproportionately affects older adults and those with underlying medical conditions, placing them at higher risk for COVID-19-related morbidity and mortality [9,10,11]. In addition, infection with SARS-CoV-2 is associated with post-acute sequelae (i.e., long COVID) that include a wide range of health issues that can occur even among patients who experienced a mild acute infection and can persist for years following initial infection [12,13,14]. According to the Household Pulse Survey, 27.6% of adults who had COVID-19 report experiencing long COVID [15]. In addition, studies have shown that patients hospitalized with COVID-19 were over twice as likely to develop hypertension compared with patients who had influenza [16], and long COVID was associated with a higher burden of disability than either heart disease or cancer [13].

Vaccination has been shown to reduce COVID-19 incidence and severity and is associated with a 30–40% lower odds of long COVID [17,18]; however, vaccine efficacy wanes with viral mutation and time since most recent dose [19,20]. Vaccination with updated formulations can help restore waning immunity and provide protection against the circulating variants [21,22]. Yet vaccination rates have decreased with each successive dose [23]. During the 2022–2023 season, vaccines were developed to counter waning immunity and broaden protection against emerging variants. Unfortunately, in the US, COVID-19 vaccination rates were low, with only ~17% of those eligible having received bivalent vaccination as of August 2023 [24], which was substantially lower than the annual influenza vaccination during the same period [25]. This raises public health concerns about suboptimal protection at the population level against COVID-19, particularly during the most recent influenza season.

Three years into the pandemic, COVID-19 remains a significant burden in comparison to other respiratory illnesses; however, many of the monitoring tools available during the early phase of the COVID-19 pandemic have been phased out, making it more difficult to track the current burden of outpatient medical encounters and hospitalizations, especially for at-risk groups. The objective of this analysis was to characterize the frequency and severity of medically attended COVID-19 and influenza during a 6-month period (1 October 2022 through 31 March 2023) that included peak influenza activity in the pediatric (0–17), adult (18–64), and older adult (65+) populations. A secondary objective was to characterize the prevalence of underlying medical conditions among patients hospitalized with COVID-19.

## 2. Materials and Methods

### 2.1. Study Design and Data Sources

We conducted a cross-sectional analysis of COVID-19 and influenza medical encounters from 1 October 2022 through 31 March 2023, a six-month period roughly covering the peak of the 2022–2023 influenza season. This study leveraged electronic health records (EHRs) from the Veradigm Health Insights Database linked to administrative claims data from Komodo Health’s Healthcare Map. The EHR dataset consists of patient records sourced from ambulatory/outpatient primary care and specialty settings and captures encounter setting, provider type, diagnoses, procedures, vitals, vaccinations, and lab results, among other variables. The insurance claims data contain inpatient, outpatient, and pharmacy claims, and only closed claims were used for this study. Patient-level files in each data source are linked by de-identified tokens created by Datavant to create a final de-identified dataset that contains no protected health information.

This integrated claims and EHR dataset have been previously characterized by Boikos et al. [26] and used previously in COVID-19 and influenza epidemiology and vaccine effectiveness research [27,28,29]. Specifically, the population captured in this dataset is pulled from across the 50 states with a regional distribution similar to the US Census Bureau data. The pediatric population is slightly under-represented, and the 65 and older population is slightly over-represented compared to the US Census data. Females are slightly over-represented compared to US Census data; however, the gender disparity aligns with previously reported differences in healthcare utilization among females and males [30].

The linked dataset has been determined to be statistically de-identified through a formal determination by a qualified expert as defined in Section §164.514(b)(1) of the Health Insurance Portability and Accountability Act Privacy Rule. As a noninterventional, retrospective database study using a certified Health Insurance Portability and Accountability Act-compliant de-identified research database, approval by an institutional review board was not required.

### 2.2. Cohort Construction

This study included all individuals in the linked dataset who had continuous claims enrollment with both pharmacy and medical benefits between 1 October 2022 and 31 March 2023 (study period) and at least one record of EHR or claims activity during that period. Codes used to identify COVID-19 and influenza cases are reported in Table S1.

### 2.3. Patient Characteristics

For each patient, we recorded age and sex at the start of the study period. Age is reported categorically in the following groups: 0–5, 6–17, 18–49, 50–64, and 65+ years old.

We also captured underlying medical conditions identified by the US Centers of Disease Control and Prevention (CDC) as associated with higher risk for severe COVID-19 [31], which were documented in the patient record during the 12 months prior to 1 October 2022. The underlying medical conditions of interest included in this analysis were attention deficit and hyperactivity disorder (ADHD), asthma, cancer, cerebral palsy, cerebrovascular disease, chronic kidney disease, chronic liver disease, chronic lung disease, congenital malformation, cystic fibrosis, dementia (only in individuals 18+), diabetes (type 1 and type 2), disability, Down syndrome, heart disease, human immunodeficiency virus (HIV), hypertension, use of select immunosuppressive medications, mental health conditions, musculoskeletal conditions, neurologic conditions, obesity (body mass index > 30), other immunocompromised condition, physical inactivity, pregnancy (only in individuals 18+), smoking (current and former; only in individuals 18+), solid organ transplant, stem cell transplant, and tuberculosis. Code sets and medication lists used to identify underlying medical conditions are listed in Table S2.

### 2.4. Study Outcomes

For each patient, we looked for encounters with a diagnosis of COVID-19 during the study period and assigned patients to one of 6 mutually exclusive categories: hospitalization with intensive care unit (ICU) admission, hospitalization without ICU, emergency department (ED) visit, urgent care visit, outpatient visit (includes but not limited to primary care visits, office visits, and telemedicine visits), or none. A patient could be included in both the COVID-19 outcomes and the influenza outcomes if they had a diagnosis of each during the study period. However, if patients had more than one encounter with a COVID-19 or influenza diagnosis during the study period, they were assigned to a single category based on the following hierarchy: ICU > hospitalization without ICU > ED > urgent care > outpatient.

This analysis was repeated for influenza diagnoses during the study period. While patients could be assigned to only one COVID-19 category and only one influenza category, they could be assigned to both a COVID-19 category and an influenza category if they had a diagnosis of each during the study period.

### 2.5. Data Analysis

All results are reported descriptively. Incidence rates were calculated per 100,000 individuals over the 6-month study period during which patients had continuous claims enrollment using the following equation: $\frac{d}{n}\times 100,000$. Ninety-five percent confidence intervals (95% CI) for the incidence rates were calculated using the normal approximation: $incidence\;rate\pm \left(1.96\times standard\;error\right),$ where the standard error is calculated assuming the crude rates have a Poisson distribution: $100,000\times \frac{\sqrt{d}}{n}$. For both calculations, *n* is the population at risk, and *d* is the number of events. All variables are categorical and reported as counts and percentages. Categorical outcomes between COVID-19 and influenza were compared using Fisher’s exact text. The data file was constructed and analyzed using SAS V9.4 (SAS, Cary, NC, USA).

## 3. Results

The final dataset included 23,526,196 individuals with at least one insurance claim or medical record and continuous claims enrollment between 1 October 2022 and 31 March 2023. Overall, 58.0% of the included population were female, and 65.7% were 18 to 64 years old at the start of the season (Table 1). In the 12 months preceding the study period, the most common underlying medical conditions were hypertension (29.0%) and obesity (21%).

### 3.1. Incidence Rate of COVID-19 or Influenza-Related Outcomes

During the study period, 5.0% (N = 1,179,960) of the study population had a medical encounter with a COVID-19 diagnosis, and 3.0% (N = 698,002) had a medical encounter with an influenza diagnosis (Table 2). The majority of both COVID-19 and influenza encounters occurred in the urgent care setting (53.5% and 69.7%, respectively). However, COVID-19 was associated with more severe outcomes as 7.0% of COVID-19 visits occurred in the hospital non-ICU setting compared to 2.6% of influenza visits, and 1.0% of COVID-19 visits occurred in the ICU setting compared to 0.4% of influenza visits.

Incidence rates of all outcomes were higher among those with COVID-19 diagnosis than among those with influenza diagnosis (Table 2), and differences were statistically significant in all outcome categories. Notably, the incidence of hospitalization without ICU was 4.6 times higher for COVID-19 than for influenza, with crude incidence (95% CI) rates of 350 (347–352) and 77 (76–78), respectively. Similarly, the incidence of ICU admission was also 4.6 times higher for COVID-19 vs. influenza, with crude incidence rates of 50 (49–50) for COVID-19 and 11 (10–11) for influenza. In addition, the incidence of outpatient visits (other) was 5.6 times higher for COVID-19 compared to influenza.

### 3.2. COVID-19 and Influenza-Related Hospitalizations by Age

Overall, between 1 October 2022 and 31 March 2023, we identified 93,888 individuals who were hospitalized with COVID-19 (with or without ICU admission) and 20,561 individuals who were hospitalized with influenza (Table 2). Roughly 12% of hospitalizations with either COVID-19 or influenza included ICU admission. In all age groups, a significantly greater number of patients were hospitalized with COVID-19 than with influenza during the study period (Table 3). Notably, the hospitalization rate for COVID-19 was 5.6 times higher compared to influenza among the 18–49 years old age group. Similarly, hospitalization rates for COVID-19 vs. influenza were 4.2 and 5 for the 50–64 and 65+ age groups, respectively.

### 3.3. COVID-19 Hospitalizations by Underlying Medical Conditions

**Figure 1:**
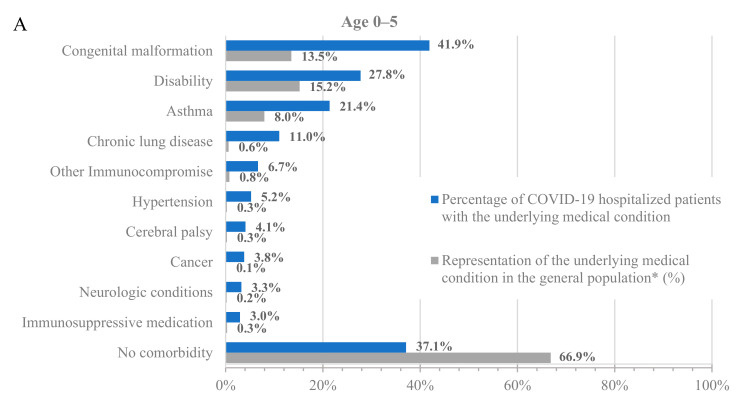

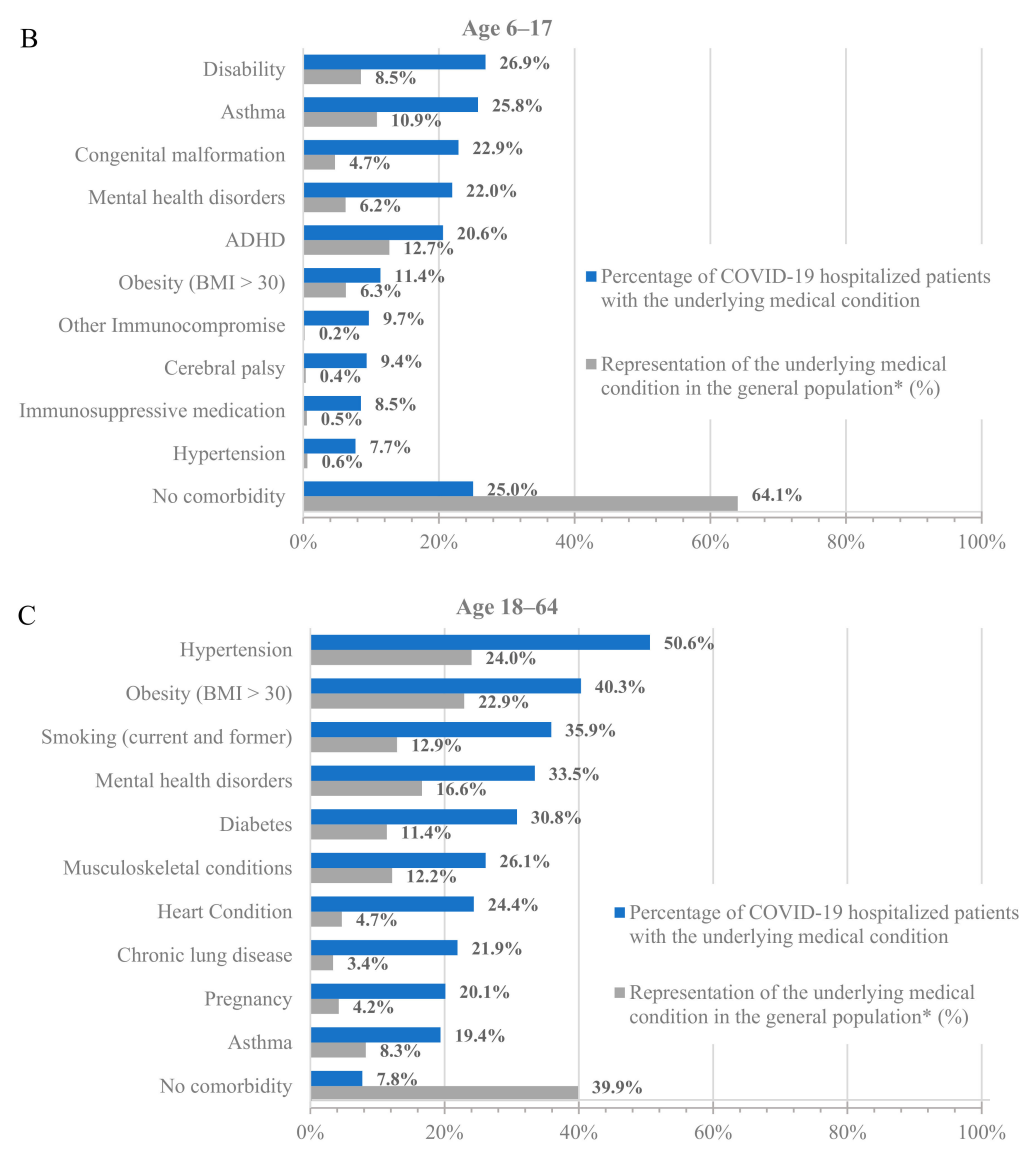

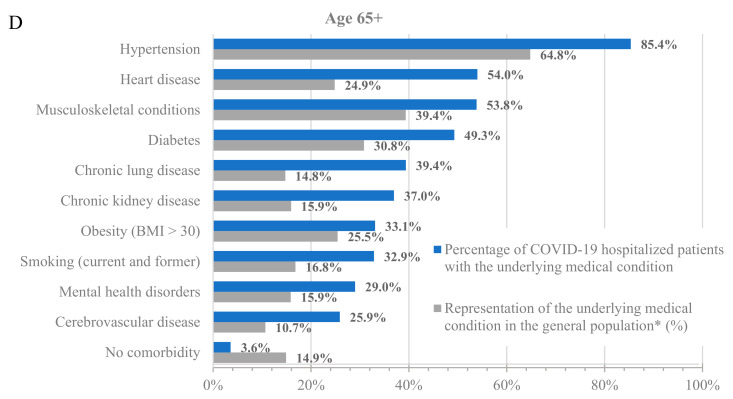
Hospitalizations by underlying medical condition by age: (**A**) 0–5 years old, (**B**) 6–17 years old, (**C**) 18–64 years old, and (**D**) 65+ years old. * As calculated in the dataset.

To further characterize the population hospitalized with COVID-19, we analyzed the percentage of hospitalized patients with underlying medical conditions (Figure 1, blue bars) and the percentage of these populations in the dataset (Figure 1, grey bars). Across all age groups, patients with underlying medical conditions were disproportionately represented among patients hospitalized with COVID-19 compared to their prevalence in the dataset (Figure 1 and Table S3). For example, 64.8% of adults at least 65 years old and 24.0% of adults 18–64 years old in the dataset had a prior diagnosis of hypertension, but 85.4% of adults at least 65 years old and 50.6% of adults 18–64 years old who were hospitalized with COVID-19 had a prior diagnosis of hypertension. Similarly, 13.5% of children 0–5 years old in the dataset had a prior diagnosis of congenital malformation, but 41.9% of children 0–5 years old who were hospitalized with COVID-19 had a prior diagnosis of congenital malformation.

Notably, almost all adults who were hospitalized for COVID-19 had at least one underlying medical condition. Specifically, only 7.8% and 3.6% of the hospitalized patients in the 18–64 and 65+ age groups, respectively, had no underlying medical condition. However, the percentage of hospitalized patients without any underlying medical conditions was much higher among the younger age groups, with 37.1% and 25% for the 0–5 and 6–17 age groups, respectively. While the prevalence of underlying medical conditions differed across age groups, the trend of an increased incidence of underlying medical conditions among patients hospitalized with COVID-19 was consistent in all groups.

Among adults at least 65 years old hospitalized for COVID-19, the most common underlying medical conditions were hypertension (85.4%), heart disease (54.0%), and musculoskeletal conditions (53.8%), such as arthritis and osteoporosis, whereas among adults 18–64 years old, the most common conditions were hypertension (50.6%), obesity (40.3%), and smoking (35.9%). Among children 0–17 years old, the most common conditions were congenital malformations (0–5: 41.9%; 6–17: 22.9%), disability (0–5: 27.8%; 6–17: 26.9%), and asthma (0–5: 21.4%; 6–17: 25.8%).

## 4. Discussion

In this analysis of over 23 million individuals who used healthcare services between 1 October 2022 and 31 March 2023 (a period that included the peak influenza activity), there were ~1.7 times as many patients with a COVID-19-related medical encounter than patients with an influenza-related medical encounter. Hospitalizations, in particular, were more common among patients with COVID-19 than among patients with influenza. Hospitalizations with COVID-19 were more common than hospitalizations with influenza in all age groups, and this difference was particularly striking among adults 18–64 and 65+ years old. Nearly all adults hospitalized with COVID-19 had at least one underlying medical condition associated with increased risk for severe outcomes. Some of the conditions that were most strongly associated with severe COVID-19-related hospitalizations, such as hypertension, diabetes, and obesity, are highly prevalent in the US adult population, including young adults as a 2020 analysis of NHANES found that 75% of all adults including 59% young adults (18–29 years old) had at least one risk factor for severe COVID-19 [32]. Importantly, 37.1% of those hospitalized at 0–5 years old and 25% of those hospitalized at 6–17 years old had no known underlying medical conditions, making it challenging to predict which children will have severe outcomes from COVID-19 infection.

The trends observed in our analysis are generally consistent with the data available for 8 October 2022 through 1 April 2023 from the CDC’s COVID-NET and FluSurv-NET databases, which track laboratory-confirmed hospitalizations from contributing hospital systems in 13 states [7,8]. Similar to our study, the incidence of hospitalizations with laboratory-confirmed COVID-19 was markedly higher (~3 times) than the incidence of hospitalizations with laboratory-confirmed influenza [7,8]. This difference is smaller than the 4.6 times higher incidence of hospitalization observed in this study but likely reflects the difference in the study methodologies, such as the differences in outcome definition (test-confirmed versus diagnosis code) and in source population (13 states versus 50 states). It is also consistent with the trends observable in the Respiratory Illnesses dashboard developed by Epic research [4].

In the CDC data for 8 October 2022 through 1 April 2023, roughly 14.6% of hospitalizations with COVID-19 included ICU admission, which is comparable to the 12.4% observed in this study [7]. In addition, previous analysis of the CDC data has found that the annual COVID-19-associated hospitalization rate from 2020–2021 was higher among children <18 years of age than the influenza-associated hospitalization rate during the prior three seasons [33]. Moreover, and consistent with our analysis, children 0–17 years old hospitalized with COVID-19 at any time were more likely to have no underlying medical conditions compared to adults at least 18 years old in the CDC data. Specifically, in the recent Advisory Committee on Immunization Practice (ACIP) COVID-19 Vaccines Work Group meeting, it was reported that 54% of children 0–17 years old hospitalized with COVID-19 had no underlying medical conditions [34]. This estimate is higher than that reported in our study, as the ACIP analysis examined a smaller number of underlying medical conditions. However, the conclusion remains consistent. Although hospitalization with COVID-19 is rare among children 0–17 years old, it is difficult to predict which children will have severe outcomes.

One notable difference between our findings and the CDC data is that in our study, individuals at least 65 years old made up only 45.2% of those hospitalized with COVID-19; however, in the CDC data for a similar time period (8 October 2022 through 1 April 2023) individuals at least 65 years old made up only 61.8% of those hospitalized with laboratory-confirmed COVID-19. This difference may be due to differences in the definition of hospitalization with COVID-19, imbalances in who gets tested for COVID-19, and fundamental differences in the sampled population of the two datasets and coverage of the Medicare population. Overall, the CDC data support our findings that during the most recent influenza season, COVID-19 presented a greater healthcare burden than influenza, regardless of age, and importantly, that burden is not only among the older adult population. Moreover, these data are consistent with other studies comparing the clinical course of COVID-19 and influenza [35,36,37].

Like influenza, the incidence of COVID-19 correlates with the arrival of new variants that evade existing immunity [38,39]; however, the arrival and transmission of new COVID-19 variants does not have an established seasonality. Therefore, it is important to acknowledge that COVID-19 diagnoses were still more frequent than influenza diagnoses, particularly among hospitalized patients, even though this study is looking at infections during a period that included the peak influenza activity.

Vaccination coverage was not measured in this analysis. Prior studies have shown that the incidence of any COVID-19 medically attended outcome is lower among vaccinated individuals compared to unvaccinated individuals [17,40]. In addition, timely administration of booster doses (either monovalent or bivalent) has been associated with a lower incidence of different COVID-19-related outcomes compared to individuals who did not receive a booster dose [22,41]. According to the CDC, the overall vaccination rates in the US with the bivalent COVID-19 vaccine as of May 2023 were 20.5% and 43.3% for the over 18 and over 65 years old age groups [24]. On the other hand, the national vaccination coverage with the influenza vaccine was 47.4% and 71.0% for the 18+ and 65+ age groups, respectively [25]. Thus, it might be that the greater COVID-19-related burden compared to influenza can be partially explained by the lower vaccination rates. As new COVID-19 variants emerge, there is a need not only to assess the effectiveness of the vaccine and update guidelines but also to provide clear evidence-based recommendations and consistent messaging on vaccines to increase vaccine confidence among HCPs and patients and ensure optimal protection against the circulating variants.

The clinical understanding of the viral mutations, at-risk population, long-term complications, and optimal vaccine schedule for COVID-19 is still evolving. Data to monitor trends in the dominant variant, test positivity rate, vaccination rates, hospitalization incidence, and characteristics of hospitalized patients exist but are located in disparate and sometimes inaccessible sources. The true utility of these data to impact clinical practice can only happen if the data are synthesized in one location with an intuitive user interface and continually refreshed so that healthcare professionals can rapidly assess the current state of COVID-19-related burden.

There are several limitations to this analysis. First, there are risks of both overestimating the incidence of medically attended COVID-19 and influenza. The use of diagnosis codes instead of positive laboratory tests risks overestimating the incidence of infection, as many respiratory infections have overlapping clinical presentations. However, during influenza season, this is likely biasing towards influenza being the preferred diagnosis. It may be biased towards COVID-19 if we are also capturing encounters for treatment of long COVID that are incorrectly coded for COVID-19. However, previous research has shown low use of the diagnosis code specific to long COVID [42]. Furthermore, this study was not designed to distinguish between patients seeking care directly for COVID-19 or influenza from those seeking care for other conditions while concurrently suffering from an infection of interest. All outcomes are interpreted as with COVID-19 or with influenza rather than for COVID-19 or influenza, as this retrospective study cannot assess causality between the infection and the patient seeking medical care.

In addition, it is possible to have COVID-19 or influenza multiple times during the same season due to the circulation of multiple COVID variants and influenza strains; however, to avoid double counting infections, which required multiple healthcare encounters, we assigned individuals to at most one COVID-19 medical encounter and one influenza medical encounter based on the highest level of care received with the respective diagnosis. This assumption may underestimate the incidence of medically attended infection.

The 6-month study period used in this analysis did not capture the full 2022–2023 influenza season as defined by the CDC. However, the peak influenza period of the 2022–2023 season is captured in this analysis and the exclusion of the tail end of the season should not be expected to significantly change the results. This is particularly true because the 2022–2023 influenza season peaked earlier than prior recent seasons [43].

This study took an inclusive approach to building code sets and, therefore, may be overestimating the prevalence of underlying medical conditions that increase the risk of severe COVID-19. For example, the code list for congenital malformation includes all ICD-10 Q codes, which include high-risk conditions like anencephaly and Down’s syndrome but also lower-risk conditions like ankyloglossia (tongue-tie). The use of a broad approach biases towards the null effect, though it likely overestimates the number of individuals with underlying medical conditions that increase the risk of severe COVID-19. Notwithstanding, this would not change the conclusion that the percentage of patients hospitalized with COVID-19 outweighs their prevalence in the dataset.

The linked data source includes only insured individuals, and the results may not be representative of the uninsured. In addition, we restricted to individuals with at least one medical record or claim between 1 October 2022 and 31 March 2023, so the population is likely to have a higher comorbidity burden than the US population as they represent people who are actively seeking care. Lastly, the pediatric population is under-represented in the linked data source as compared to the US population [26]. Therefore, the pediatric infection burden might be underestimated when compared to older age cohorts.

## 5. Conclusions

During the 2022–2023 influenza season, nearly three years after the start of the pandemic, medical encounters with a diagnosis of COVID-19 were more common than medical encounters with a diagnosis of influenza. In particular, hospitalizations with COVID-19 were more common than hospitalizations with influenza in all age subgroups examined. This may be explained in part by the lower vaccination rates with the COVID-19 vaccine. Vaccination against COVID-19 remains the most effective intervention to protect against severe outcomes, especially for those who are at higher risk of developing serious complications, including children, older adults, and those with chronic conditions.

## Figures and Tables

**Table 1:** Population Characteristics.

	All Patients	
	N = 23,526,196	
Characteristics	N	%
Female sex	13,634,220	58.0%
Age		
0–5	506,690	2.2%
6–17	2,794,168	11.9%
18–49	9,717,350	41.3%
50–64	5,737,529	24.4%
65+	4,770,459	20.3%
Underlying medical conditions ^a^		
Hypertension	6,824,263	29.0%
Obesity (body mass index > 30)	4,942,037	21.0%
Musculoskeletal conditions	3,772,098	16.0%
Mental health disorders	3,504,546	14.9%
Diabetes	3,254,728	13.8%
Smoking (current and former)	2,802,473	11.9%
Asthma	1,998,127	8.5%
Heart disease	1,918,234	8.2%
Chronic lung disease	1,238,751	5.3%
ADHD	1,139,822	4.8%
Chronic kidney disease	1,112,167	4.7%
Cancer	991,733	4.2%
Cerebrovascular disease	807,423	3.4%
Congenital malformation	736,570	3.1%
Immunosuppressive medications	719,139	3.1%

^a^ Underlying medical conditions impacting less than 3% of the overall population are not listed.

**Table 2:** COVID-19 and influenza medical encounters between 1 October 2022 and 31 March 2023.

Outcome	COVID-19			Influenza			Incidence Ratio of COVID-19 to Influenza
	N = 1,179,960			N = 698,002			
	N	% ^a^	Incidence ^b^ (95% CI)	N	% ^a^	Incidence ^b^ (95% CI)	
Outpatient visits (other)	283,213	24.0% *	1204 (1199–1208)	50,835	7.3%	216 (214–218)	5.6
Urgent care visit	631,356	53.5% *	2684 (2677–2690)	486,406	69.7%	2068 (2062–2073)	1.3
ED visit	171,503	14.5% *	729 (726–732)	140,200	20.1%	596 (593–599)	1.2
Hospitalization ^c^	82,234	7.0% *	350 (347–352)	18,032	2.6%	77 (76–78)	4.6
ICU	11,654	1.0% *	50 (49–50)	2529	0.4%	11 (10–11)	4.6

CI, confidence interval; ED, emergency department; ICU, intensive care unit. ^a^ Percents calculated per total number of COVID-19 or influenza cases. ^b^ Incidence calculated as per 100,000 people per 6-month season among the 23,526,196 individuals with continuous claims enrollment for that period, ^c^ not including ICU. * *p*-value < 0.001, COVID-19 versus influenza.

**Table 3:** Hospitalizations ^a^ with a diagnosis of COVID-19 or influenza across age groups between 1 October 2022 and 31 March 2023.

Age Group	COVID-19	Influenza	Count Ratio of COVID-19 vs. Influenza
	N = 93,888	N = 20,561	
	N	N	
Pediatrics			
0–5	706 *	564	1.3
6–17	1529 *	1260	1.2
Adults			
18–49	26,242 *	4693	5.6
50–64	22,947 *	5529	4.2
Older Adults			
65+	42,464 *	8515	5

^a^ Inclusive of intensive care unit admission. * *p*-value < 0.001, COVID-19 versus influenza.

## Data Availability

The data that support the findings of this study were used under license from Veradigm and Komodo Health. Due to data use agreements and their proprietary nature, restrictions apply regarding the availability of the data. Further information is available from the corresponding author.

## Supplementary Materials

The following supporting information can be downloaded at: https://www.mdpi.com/article/10.3390/diseases12010016/s1, Table S1: COVID-19 and influenza code lists; Table S2: Underlying medical conditions code lists; Table S3. Comorbidity burden among patients hospitalized with COVID-19.
