# Supplementary material for: Comparison of COVID-19 and Influenza-Related Outcomes in the United States during Fall–Winter 2022–2023: A Cross-Sectional Retrospective Study
Source: Diseases. 2024 Jan 3;12(1):16. doi: 10.3390/diseases12010016 (PMC10814040; doi:10.3390/diseases12010016)
Supplement: Supplementary file 1 [file diseases-12-00016-s001.zip › Table S1.pdf]

Table S1: COVID-19 and influenza code lists

**Condition**	**Type**	**Codes**
COVID-19	ICD-10-CM Dx	U071, B342, B9729, Z8616, J1282
	ICD10	U072
	SNOMED	840533007, 840539006, 1119302008, 119731000146105, 119741000146102, 119751000146104, 119981000146107, 1240521000000100, 1240531000000100, 1240541000000100, 1240561000000100, 1240581000000100, 674814021000119000, 866151004, 866152006, 870577009, 870588003, 870589006, 870590002, 870591003, 871562009, 1240411000000100, 840536004, 840534001
Influenza	ICD-10-CM Dx	J09, J09x, J09X1, J09X2, J09X3, J09X9, J10, J100, J1000, J1001, J1008, J101, J102, J108, J1081, J1082, J1083, J1089, J11, J110, J1100, J1108, J111, J112, J118, J1181, J1182, J1183, J1189
