# Supplementary material for: Comparison of COVID-19 and Influenza-Related Outcomes in the United States during Fall–Winter 2022–2023: A Cross-Sectional Retrospective Study
Source: Diseases. 2024 Jan 3;12(1):16. doi: 10.3390/diseases12010016 (PMC10814040; doi:10.3390/diseases12010016)
Supplement: Supplementary file 1 [file diseases-12-00016-s001.zip › Table S3.pdf]

Table S3: Comorbidity burden among patients hospitalized with COVID-19

	Age 0-5			
	Hospitalized* patients		All individuals in dataset	
	N =	706	N =	506,690
	N	%	N	%
No comorbidity	262	37.1%	338,785	66.9%
Cancer	27	3.8%	668	0.1%
Cerebrovascular disease	15	2.1%	1,204	0.2%
Chronic kidney disease	11	1.6%	422	0.1%
Chronic lung disease	78	11.0%	3,160	0.6%
Chronic liver disease	<10	N/A	58	0.0%
Cystic fibrosis	<10	N/A	413	0.1%
Diabetes	<10	N/A	710	0.1%
Disability	196	27.8%	77,142	15.2%
Heart Condition	18	2.5%	1,062	0.2%
HIV	<10	N/A	29	0.0%
Mental health disorders	<10	N/A	702	0.1%
Dementia	N/A	N/A	N/A	N/A
Obesity (BMI > 30)	10	1.4%	7,966	1.6%
Other Immunodeficiencies	47	6.7%	3,962	0.8%
Pregnancy	N/A	N/A	N/A	N/A
Physical inactivity	<10	N/A	183	0.0%
Smoking (current and former)	N/A	N/A	N/A	N/A
Solid organ transplant	<10	N/A	120	0.0%
Tuberculosis	<10	N/A	30	0.0%
Immunosuppressive medication	21	3.0%	1,606	0.3%
Asthma	151	21.4%	40,489	8.0%
Neurologic conditions	23	3.3%	1,166	0.2%
Musculoskeletal conditions	<10	N/A	377	0.1%
Hypertension	37	5.2%	1,445	0.3%
ADHD	<10	N/A	4,291	0.8%
Cerebral palsy	29	4.1%	1,480	0.3%
Congenital malformation	296	41.9%	68,519	13.5%
Down syndrome	14	2.0%	1,242	0.2%
Stem cell transplant	<10	N/A	67	0.0%

*cell counts of 1-9 are reported as <10 to ensure data is deidentified. Zeros are reported as zeros.
ADHD, attention deficit hyperactivity disorder; BMI, body mass index; HIV, human immunodeficiency virus

	Age 6-17			
	Hospitalized* patients		All individuals in dataset	
	N =	1,529	N =	2,794,168
	N	%	N	%
No comorbidity	383	25.0%	1,789,787	64.1%
Cancer	98	6.4%	6,266	0.2%
Cerebrovascular disease	48	3.1%	3,350	0.1%
Chronic kidney disease	43	2.8%	2,972	0.1%
Chronic lung disease	108	7.1%	5,427	0.2%
Chronic liver disease	10	0.7%	1,136	0.0%
Cystic fibrosis	14	0.9%	1,214	0.0%
Diabetes	69	4.5%	20,212	0.7%
Disability	411	26.9%	237,885	8.5%
Heart Condition	53	3.5%	3,976	0.1%
HIV	<10	N/A	242	0.0%
Mental health disorders	336	22.0%	174,464	6.2%
Dementia	N/A	N/A	N/A	N/A
Obesity (BMI > 30)	174	11.4%	175,954	6.3%
Other Immunodeficiencies	148	9.7%	6,162	0.2%
Pregnancy	N/A	N/A	N/A	N/A
Physical inactivity	<10	N/A	1,871	0.1%
Smoking (current and former)	N/A	N/A	N/A	N/A
Solid organ transplant	30	2.0%	1,250	0.0%
Tuberculosis	0	0.0%	137	0.0%
Immunosuppressive medication	130	8.5%	14,878	0.5%
Asthma	394	25.8%	303,733	10.9%
Neurologic conditions	56	3.7%	5,672	0.2%
Musculoskeletal conditions	26	1.7%	7,961	0.3%
Hypertension	118	7.7%	17,596	0.6%
ADHD	315	20.6%	354,833	12.7%
Cerebral palsy	143	9.4%	10,353	0.4%
Congenital malformation	350	22.9%	130,976	4.7%
Down syndrome	16	1.0%	5,749	0.2%
Stem cell transplant	12	0.8%	555	0.0%

*cell counts of 1-9 are reported as <10 to ensure data is deidentified. Zeros are reported as zeros.
ADHD, attention deficit hyperactivity disorder; BMI, body mass index; HIV, human immunodeficiency virus

	Age 18-64			
	Hospitalized* patients		All individuals in dataset	
	N =	49,189	N =	15,454,879
	N	%	N	%
No comorbidity	3,819	7.8%	6,164,086	39.9%
Cancer	4,312	8.8%	428,136	2.8%
Cerebrovascular disease	5,063	10.3%	292,406	1.9%
Chronic kidney disease	7,805	15.9%	347,900	2.3%
Chronic lung disease	10,787	21.9%	525,818	3.4%
Chronic liver disease	2,306	4.7%	121,708	0.8%
Cystic fibrosis	89	0.2%	3,910	0.0%
Diabetes	15,147	30.8%	1,762,550	11.4%
Disability	1,131	2.3%	153,525	1.0%
Heart Condition	11,987	24.4%	727,435	4.7%
HIV	751	1.5%	78,027	0.5%
Mental health disorders	16,459	33.5%	2,572,057	16.6%
Dementia	1,605	3.3%	52,931	0.3%
Obesity (BMI > 30)	19,831	40.3%	3,543,618	22.9%
Other Immunodeficiencies	2,187	4.4%	85,959	0.6%
Pregnancy	9,901	20.1%	655,765	4.2%
Physical inactivity	139	0.3%	12,625	0.1%
Smoking (current and former)	17,657	35.9%	1,999,052	12.9%
Solid organ transplant	1,320	2.7%	36,212	0.2%
Tuberculosis	74	0.2%	4,798	0.0%
Immunosuppressive medication	3,952	8.0%	448,519	2.9%
Asthma	9,546	19.4%	1,278,488	8.3%
Neurologic conditions	1,782	3.6%	117,760	0.8%
Musculoskeletal conditions	12,847	26.1%	1,885,491	12.2%
Hypertension	24,891	50.6%	3,713,659	24.0%
ADHD	2,402	4.9%	757,056	4.9%
Cerebral palsy	379	0.8%	27,729	0.2%
Congenital malformation	3,338	6.8%	392,380	2.5%
Down syndrome	95	0.2%	10,815	0.1%
Stem cell transplant	157	0.3%	4,474	0.0%

*cell counts of 1-9 are reported as <10 to ensure data is deidentified. Zeros are reported as zeros.
ADHD, attention deficit hyperactivity disorder; BMI, body mass index; HIV, human immunodeficiency virus

	Age 65+			
	Hospitalized* patients		All individuals in dataset	
	N =	42,464	N =	4,770,459
	N	%	N	%
No comorbidity	1,527	3.6%	711,992	14.9%
Cancer	8,263	19.5%	556,663	11.7%
Cerebrovascular disease	10,995	25.9%	510,463	10.7%
Chronic kidney disease	15,699	37.0%	760,873	15.9%
Chronic lung disease	16,731	39.4%	704,346	14.8%
Chronic liver disease	1,517	3.6%	69,276	1.5%
Cystic fibrosis	13	0.0%	441	0.0%
Diabetes	20,931	49.3%	1,471,256	30.8%
Disability	319	0.8%	14,801	0.3%
Heart Condition	22,935	54.0%	1,185,761	24.9%
HIV	241	0.6%	14,711	0.3%
Mental health disorders	12,325	29.0%	757,323	15.9%
Dementia	8,802	20.7%	314,610	6.6%
Obesity (BMI > 30)	14,063	33.1%	1,214,499	25.5%
Other Immunodeficiencies	1,873	4.4%	74,559	1.6%
Pregnancy	<10	N/A	185	0.0%
Physical inactivity	143	0.3%	5,943	0.1%
Smoking (current and former)	13,963	32.9%	803,421	16.8%
Solid organ transplant	776	1.8%	17,103	0.4%
Tuberculosis	45	0.1%	2,303	0.0%
Immunosuppressive medication	3,686	8.7%	254,136	5.3%
Asthma	5,961	14.0%	375,417	7.9%
Neurologic conditions	2,803	6.6%	113,066	2.4%
Musculoskeletal conditions	22,864	53.8%	1,878,269	39.4%
Hypertension	36,246	85.4%	3,091,563	64.8%
ADHD	209	0.5%	23,642	0.5%
Cerebral palsy	96	0.2%	4,372	0.1%
Congenital malformation	2,306	5.4%	144,695	3.0%
Down syndrome	13	0.0%	357	0.0%
Stem cell transplant	77	0.2%	2,307	0.0%

*cell counts of 1-9 are reported as <10 to ensure data is deidentified. Zeros are reported as zeros.
ADHD, attention deficit hyperactivity disorder; BMI, body mass index; HIV, human immunodeficiency virus
